# A Flexible Model of HIV-1 Latency Permitting Evaluation of Many Primary CD4 T-Cell Reservoirs

**DOI:** 10.1371/journal.pone.0030176

**Published:** 2012-01-24

**Authors:** Kara G. Lassen, Andrew M. Hebbeler, Darshana Bhattacharyya, Michael A. Lobritz, Warner C. Greene

**Affiliations:** 1 Gladstone Institute of Virology and Immunology, San Francisco, California, United States of America; 2 Department of Medicine, University of California San Francisco, San Francisco, California, United States of America; 3 Department of Microbiology and Immunology, University of California San Francisco, San Francisco, California, United States of America; 4 Case Western Reserve University School of Medicine, Cleveland, Ohio, United States of America; University Hospital Zurich, Switzerland

## Abstract

Latently infected cells form the major obstacle to HIV eradication. Studies of HIV latency have been generally hindered by the lack of a robust and rapidly deployable cell model that involves primary human CD4 T lymphocytes. Latently infected cell lines have proven useful, but it is unclear how closely these proliferating cells recapitulate the conditions of viral latency in non-dividing CD4 T lymphocytes *in vivo*. Current primary lymphocyte models more closely reflect the *in vivo* state of HIV latency, but they are limited by protracted culture periods and often low cell yields. Additionally, these models are always established in a single latently infected cell type that may not reflect the heterogeneous nature of the latent reservoir. Here we describe a rapid, sensitive, and quantitative primary cell model of HIV-1 latency with replication competent proviruses and multiple reporters to enhance the flexibility of the system. In this model, post-integration HIV-1 latency can be established in all populations of CD4 T cells, and reactivation of latent provirus assessed within 7 days. The kinetics and magnitude of reactivation were evaluated after stimulation with various cytokines, small molecules, and T-cell receptor agonists. Reactivation of latent HIV proviruses was readily detected in the presence of strong activators of NF-κB. Latently infected transitional memory CD4 T cells proved more responsive to these T-cell activators than latently infected central memory cells. These findings reveal potentially important biological differences within the latently infected pool of memory CD4 T cells and describe a flexible primary CD4 T-cell system to evaluate novel antagonists of HIV latency.

## Introduction

Within days after initial infection, HIV-1 establishes a persistent latent reservoir in resting CD4 T cells and possibly other cell types in all infected subjects [1], [2], [3]. Latently infected cells harbor integrated HIV-1 proviral DNA but are otherwise indistinguishable from uninfected cells. Although they are rare *in vivo,* their longevity and resistance to antiretroviral therapy make them a major barrier to HIV-1 eradication [4], [5], [6].

Even studying latently infected cells from HIV-infected subjects is challenging. These cells are very rare in the blood, and there are no methods to enrich them. One approach for attacking the latent reservoir is to use activating compounds that specifically induce transcription of the latent provirus and translation of HIV proteins but that are not toxic to uninfected CD4 T cells. To identify such activators and to better understand the biological underpinnings of HIV latency, a robust, flexible, and easy to construct model of HIV latency in primary CD4 T cells is urgently needed.

To date, the best-characterized models of HIV latency involve immortalized T-cell lines [7]. These systems have improved our understanding of the relationship between T-cell stimulation and proviral reactivation and the dynamic changes in chromatin structure and transcription factor binding that accompany HIV LTR reactivation [8], [9], [10], [11], [12]. However, these they are imperfect surrogates: they do not recapitulate the non-dividing G_0_ state of resting CD4 T cells *in vivo* [1], [4]. Primary resting CD4 T cells provide the optimal intracellular milieu for establishing latency but are inefficiently infected *in vitro*, since HIV is impaired during reverse transcription [13] and integration [10], [14]. Most primary cell models use one or more rounds of cellular stimulation to remove these blocks, followed by HIV infection during the return to a resting state [15], [16], [17], [18], [19]. Unfortunately, although latently infected non-dividing T cells are generated, the process often takes several weeks or months of continuous culture. Many primary cell models also require cell sorting techniques to achieve pure populations of cells before or after infection, a process that greatly reduces the total yield of cells [20], [21]. These features make it difficult to execute large-scale screening for agents that could reactivate and eliminate latent proviruses.

Recent reports suggest latent reservoir *in vivo* might be more complex than thought. In one study of patients on antiretroviral therapy with clinically undetectable viral levels, two cellular reservoirs were detected. One decayed with antiretroviral therapy, and one did not [22]. In a second study, proviral DNA was preferentially detected within two different memory CD4 T-cell subpopulations, specifically central memory and transitional memory cells [23]. Although central memory T cells typically harbor a larger proportion of the latent proviruses, the transitional memory cells appear to live longer and are continually renewed by cytokine-induced homeostatic proliferation [23]. It is unknown approaches aimed at purging latent proviruses will be as effective in these different memory cell populations. In addition, since current models of HIV-1 latency involve one or more rounds of cellular stimulation, it is difficult to know if latency is reproducibly established in both memory cell types as it is *in vivo*. A model of HIV-1 latency that allows to examine latency in both memory CD4 T-cell subpopulations would be very useful.

In the model of HIV-1 latency originally described by the O'Doherty laboratory, resting CD4 T cells are directly infected by spinoculation [20], [24]. With its high levels of virion attachment, a proportion of viruses likely complete reverse transcription and integration [24]. Post-integration latency is established in these spinoculated cells within 72 h in all CD4 T-cell subsets, including both naive and memory T cells [25], [26], [27]. Latent proviruses are activated after an additional 72 h of cellular stimulation [24], indicating that latency can be established and reactivation assessed within 6 days. The speed and reproducibility of this system made it an ideal starting point for developing an even more dynamic primary CD4 T-cell model of HIV latency suitable for screening of reactivating agents.

Using novel reporter viruses, we describe an improved version of this primary CD4 T-cell model that can be used to study latency in all subsets of CD4 T cells. We specifically evaluated differences in HIV latency in central and transitional memory CD4 T cells.

## Methods

### Ethics Statement

This study was conducted according to the principles expressed in the Declaration of Helsinki. All individuals provided written informed consent for the collection of samples and subsequent analysis as approved by the Institutional Review Board of Stanford University Blood Bank.

#### Construction of NL4-3 luciferase and NL4-3 mCherry:Luc

In addition to the green fluorescent protein (GFP) reporter virus that measures the number of cells in which the latent HIV provirus is successfully reactivated, we created a luciferase-expressing virus that measures overall levels of transcriptional reactivation of latent HIV. A fully infectious molecular clone of NL4-3 expressing firefly luciferase from the native LTR was prepared, essentially as described below,for the replication-defective pseudotyping vector pNL-Luc-E^−^R^−^. Both pNL-Luc-E^−^R^−^ and the fully infectious molecular clone, pNL4-3, were obtained from the AIDS Research and Reference Reagent Program. pNL-Luc-E^−^R^−^ was originally generated by transposition of the firefly luciferase gene from the molecular clone pHXB-Luc [28] into pNL4-3 between the BamHI (nt 8021) and XhoI sites (nt 8443) within the *nef* coding region [29]. The BamHI-XhoI fragment of pNL-Luc-E^−^R^−^ was shuttled into pNL4-3 to yield an *env*+/*vpr*+ vector that, when transfected, produces viruses capable of multiple rounds of infection and luciferase driven from the viral LTR. We also prepared an HIV dual reporter vector expressing mCherry and luciferase to simultaneously measure the number of cells containing reactivated latent provirus and the overall strength of the viral transcriptional response in these cells. To generate a fully infectious molecular clone expressing both of these reporters, firefly luciferase was inserted in place of the puromycin resistance gene in a modified pSicoR lentiviral expression vector termed pSicoRMS2 (a kind gift of Matt Spindler and Bruce Conklin, Gladstone Institute of Cardiovascular Disease). This vector contains an EF-1 alpha–driven mCherry:Puromycin cassette in which mCherry and puromycin are separated by a picornavirus-derived ribosomal skipping T2A sequence. The T2A sequence (ccccgggagggcagaggaagtcttctaacatgcggtgacgtggaggagaatcccggccctcga) allows balanced production of the two flanking gene products [30], [31]. The firefly luciferase gene was subcloned in place of puromycin with XmaI and EcoRI. Clones were then tested for mCherry and luciferase expression after transfection of 293T cells. Mcherry:T2A:luciferase was amplified using PCR primers containing 5′ and 3′ sequences from the pNLENG1 vector (NL4-3 GFP). This amplicon was digested with BamHI and SalI and inserted into the pNLENG1 vector backbone at the unique BamHI and XhoI sites. The XhoI site was destroyed in the cloning process, resulting in an S34C mutation in Nef.

#### Isolation of CD4 T cells

Peripheral blood mononuclear cells (PBMC) were isolated by Ficoll-Hypaque density gradient centrifugation of buffy coats from HIV-seronegative donors (Stanford University Medical Center Blood Bank). PBMCs were immediately processed to isolate CD4 T cells or maintained in RPMI 1640 medium supplemented with 10% FBS and penicillin/streptomycin for up to 24 h before cellular isolation. Longer periods of culture before cellular isolation were avoided to eliminate higher levels of nonspecific cellular activation. Total CD4 T cells were isolated by negative selection, according to manufacturer's protocol, with the EasySep CD4+ T-cell Enrichment Kit (Stem Cell Technologies). Memory CD4 T cells were isolated using EasySep Memory CD4+ T-cell Enrichment Kit, according to manufacturers protocol (Stem Cell Technologies). Resting memory CD4 T cells were isolated by EasySep custom cell purification kit that depleted cells expressing CD8, CD14, CD16, CD19, CD20, CD41, CD56, GlyA, CD123, CD25, HLA-DR, and CD69. Isolated CD4 T cells were cultured in RPMI as described above at a concentration of 1×10^6^ cells/ml for 2–48 h before HIV infection.

#### Spinoculation of CD4 T cells

CD4 T cells were counted, collected as pellets by centrifugation at 200× *g* for 10 min at room temperature, and resuspended in the appropriate volume of concentrated viral supernatant. Typically, 50–200 ng of p24^Gag^ per 4×10^5^ CD4 T cells were used. Spinoculations were performed in 96-well V-bottom plates with up to 5×10^5^ CD4 T cells per well; 15-ml Falcon conical tubes were used for larger quantities of cells (up to 1×10^7^ CD4 T cells/tube). All spinoculations were performed in volumes of 200 µl or less. Cells and virus were centrifuged at 1200× *g* for 1.5–2 h at room temperature. After spinoculation, cells were pooled and cultured at a concentration of 1×10^6^ cells/ml in RPMI 1640 containing 10% FCS and supplemented with 5 µM saquinavir for 3 days to prevent residual spreading infection. Saquinavir was obtained through the AIDS Research and Reference Reagent Program, Division of AIDS, NIAID, NIH.

### Flow cytometry

To determine the presence of memory cell subpopulations or for cell sorting of memory cell populations, cells were stained with CD45RA-APC-Cy7 (1∶40), CCR7-PE-Cy7 (1∶40), CD27-APC (1∶5), and either CD45RO-FITC (1∶20) for cells infected or to be infected with NL4-3 mCherry:Luc or CD45RO-PE for cells infected or to be infected with NL4-3 GFP. Cells were stained for 30 min at 4°C, washed two times with PBS containing 2% serum, and fixed in 2% paraformaldehyde for flow cytometric analysis or left unfixed for cell sorting. Cells were analyzed with a Becton Dickinson (BD) LSRII instrument or sorted with a BD FACS Aria II flow cytometer.

### Reactivation of latent HIV-1 provirus

Cells were counted and collected as pellets by centrifugation at 200× g for 10 min. Cells were then plated in 96-well U-bottom plates at concentrations of 2.5×10^5^–1×10^6^/200 µl in the presence of 30 µM raltegravir and the indicated activator. Raltegravir (donated by Merck & Co.) was obtained from the AIDS Research and Reference Reagent Program, Division of AIDS, NIAID, NIH (Cat # 11680). Unless otherwise indicated, cells were cultured either in medium alone or stimulated with 200 nM PMA (Sigma), 1.5 µM ionomycin (Sigma), 10 µg/ml phytohemagglutinin (PHA) (Sigma), 10 ng/ml TNF-α, anti-CD3+anti-CD28 beads at a ratio of 1∶1 (Invitrogen), 62.5 ng/ml IL-7 (R&D Systems), or 12.5 ng/ml interleukin (IL)-15 (R&D Systems). TSA (Sigma), prostratin (Sigma), hexamethylene bisacetamide (HMBA) (Sigma), VPA (Sigma), and SAHA (NCI Chemical Carcinogen Repository, Midwest Research Institute) were tested at the indicated concentrations. Cells were harvested at the indicated times after spinoculation, washed one time with PBS, and lysed in 65 µl of Glo Lysis Buffer (Promega) or fixed in 2% paraformaldehyde for GFP or mCherry analysis. For luciferase samples, after 15 min of lysis, the luciferase activity in cell extracts was quantified with a BD Monolight Luminometer after mixing 50 µl of lysate with 50 µl of substrate (Luciferase Assay System-Promega). Relative light units were normalized to protein content determined by BCA assay (Pierce).

### Integration Assay

HIV integration analysis was performed as described [32]. HIV integration events were normalized to RNaseP (Applied Biosystems) to determine HIV integration events/cell.

## Results

### Rapid generation of latently infected CD4 T-cells *ex vivo*

**Figure 1:**
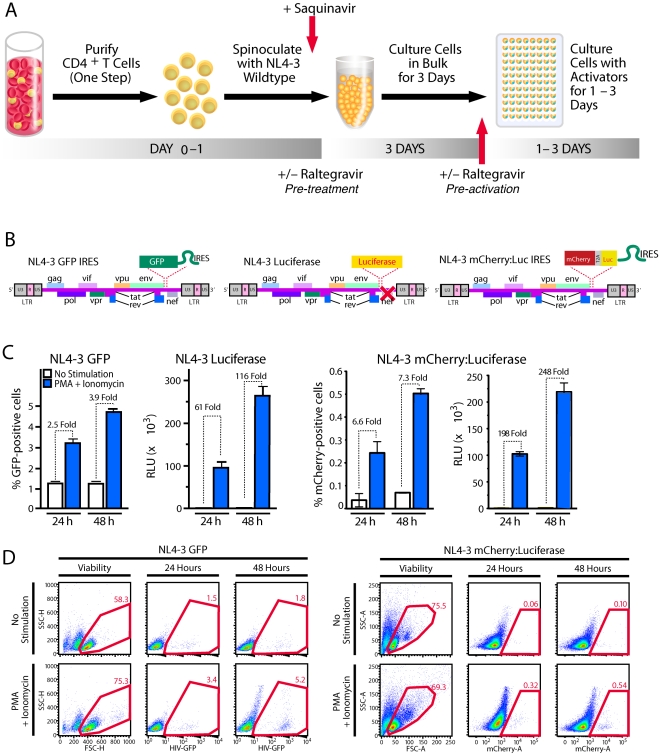
Establishing postintegration HIV-1 latency in primary CD4 T cells and reactivating virus. (A) Production of a primary cell model of HIV-1 latency. Total CD4 T cells were isolated from PBMC with a single-step negative-selection procedure with magnetic beads to remove unwanted cell subpopulations. Within 24 h, isolated cells were spinoculated at 1200× *g* for 2 h at room temperature with viral supernatants corresponding to NL4-3 GFP IRES Nef, NL4-3 Luciferase, or NL4-3 mCherry:Luciferase viruses as schematically depicted in (B). After spinoculation, cells were placed in medium containing 5 µM saquinavir and cultured for 3 days. Cells are then counted and plated in 96-well plates in medium containing 30 µM raltegravir and various stimulators. (C) Reactivation profiles of cells latently infected with NL4-3 GFP IRES Nef, NL4-3 Luciferase, or NL4-3 mCherry:Luciferase. Latently infected cells were cultured with medium alone or medium containing 200 nM PMA and 1.5 µM ionomycin and harvested after 24 or 48 hours of culture. GFP- or mCherry-expressing cells were quantified by flow cytometry, and the percentage of GFP^+^ or mCherry cells was calculated based on uninfected controls. Luciferase levels are reported as relative light units (RLU) and have been normalized to total protein content in cell lysates to control for different cell proliferation rates. All samples were analyzed in triplicate with error bars representing +/− SD. Results are representative of those obtained in analyses of at least 10 independent donors with each virus. (D) Flow cytometric gating and analysis of cells latently infected with NL4-3 GFP IRES Nef or NL4-3 mCherry:Luciferase 24 or 48 hours after stimulation with PMA and ionomycin. Forward scatter versus side scatter plots show cells infected with virus and left unstimulated or stimulated for 24 h.

To establish latently infected primary CD4 T cells, we used spinoculation to efficiently deliver large quantities of virions to resting CD4 T cells [24]. The original model of HIV-1 latency developed in the O'Doherty laboratory, like many others, involved fluorescence-activated cell sorting (FACS) to isolate highly purified resting CD4 T cells [24]. To simplify the cell-purification process, increase cell yield, and leave the desired cell population “untouched,” we used a single-step negative-selection strategy. Specifically, cells were incubated with antibody-bound magnetic beads to isolate total CD4 T cells (CD3+CD4+) or memory (CD4+CD45RO+) CD4 T cells from the peripheral blood of uninfected human volunteers. Typically, the CD4 T-cell yield was 25–30% (total CD4) or 10–15% (memory CD4) of the total PBMC with purities of 97–99% (Figure 1A, Figure S1A).

This assay required a marker to monitor the number of cells in which latent proviruses were reactivated. A full-length, replication-competent HIV expressing EGFP in the Nef position with Nef expressed under the control of an IRES element (NL4-3 GFP) (Figure 1B) [33], [34] provided useful information about the number of reactivated cells but little quantitative data on the absolute levels of reporter protein production within these cells. We generated a replication-competent version of NL4-3 NL4-3 Luciferase and a third reporter expressing mCherry and firefly luciferase separated by a T2A ribosomal skip sequence [30], [31] (Figure 1B). In the latter reporter virus, mCherry and luciferase are expressed from the same LTR-driven mRNA and translated as separate proteins in equivalent quantities, and Nef is expressed under the control of an IRES element. This latter virus permitted simultaneous assessment of the number of cells in which latent virus was reactivated (mCherry) and the strength of viral reactivation in the cells (luciferase). Stimulation with PMA and ionomycin produced 2–3.9-fold more GFP-expressing cells than uninduced cells, a 6.6–7.3-fold increase in mCherry-positive cells, and a 61–248-fold increase in luciferase activity, depending on the use of single or dual reporter viruses and the time selected for analysis (Figure 1C,D).

Reactivation of latent proviruses was easily detectable after only 24 h of stimulation with PMA and ionomycin. Higher levels were detected at 48 h (Figure 1C,D). Consistent results were obtained when more highly purified memory CD4 T cells or resting memory CD4 T cells were used, indicating that more extensive purification steps are not necessary to obtain physiologically relevant data (Figure S1B,C). Finally, this reactivation assay proved highly reproducible, based on the analysis of 10 independent donors who exhibited similar profiles with variation observed only at the level of proviral reactivation and background under unstimulated conditions (Figure S2).

### Latently infected CD4 T cells harbor integrated HIV-1 DNA

**Figure 2:**
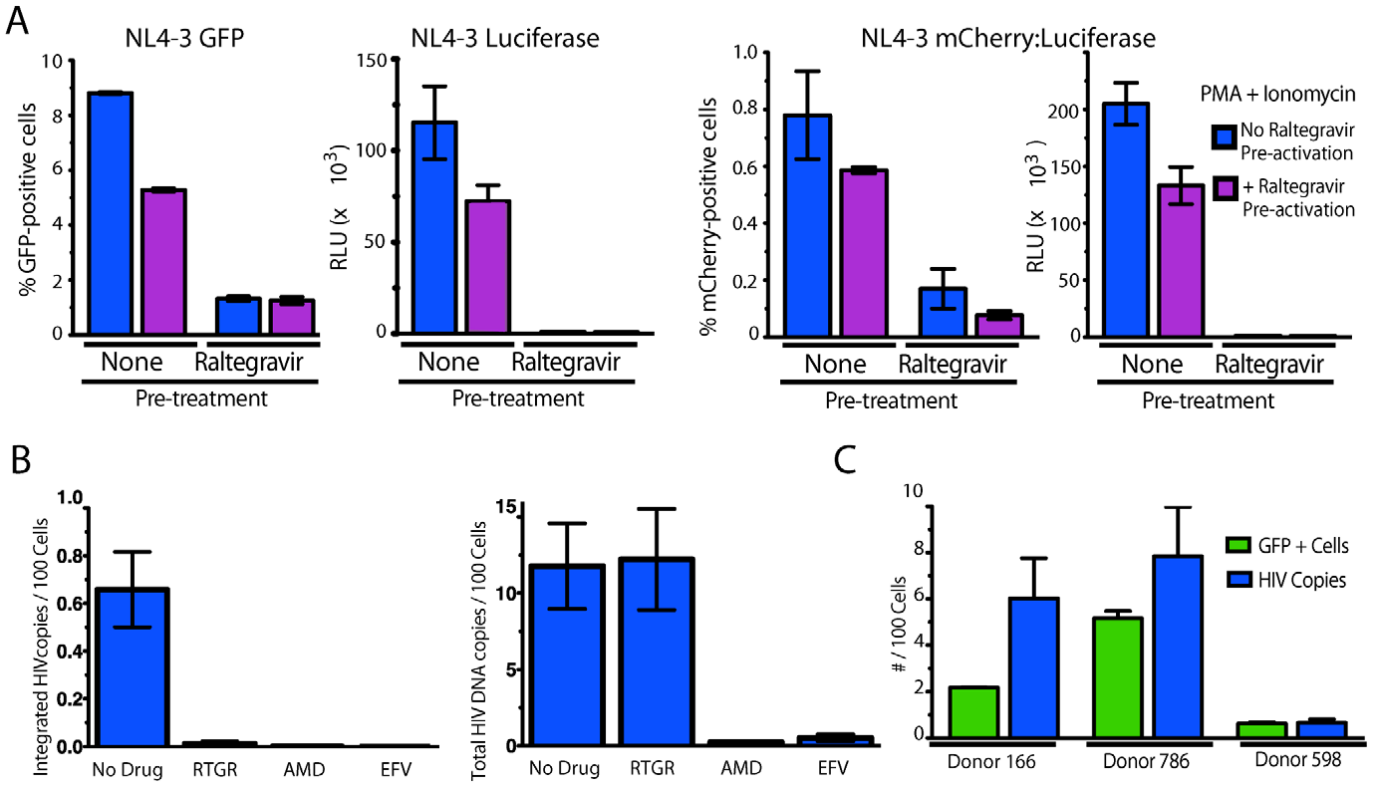
Latently infected cells harbor integrated proviruses. (A) Cells were cultured in the presence (pre-treatment) or absence (no pre-treatment) of 30 µM raltegravir that was added immediately after spinoculation. After 3 days, cells were washed and stimulated with PMA+ionomycin in the presence or absence of 30 µM raltegravir (pre-activation). Results are representative of data obtained using three independent donors with each reporter virus. Error bars represent +/−SD of triplicate experiments. (B) CD4 T cells were isolated and pre-treated with 30 µM raltegravir, 250 nM AMD3100, 100 nM Efavirenz, or medium alone for 30 min before spinoculation. Cells were spinoculated and then cultured in the presence or absence of each antiretroviral drug. Three days after spinoculation, total DNA was isolated from the cells, and levels of HIV integration were determined by Alu-gag qPCR (left panel) or levels of total HIV DNA were determined by gag qPCR (right panel). Viral integration levels were compared in cultures incubated in medium alone versus in the presence of raltegravir (RTGR), AMD3100 (AMD), or Efavirenz (EFV) antiviral drugs to confirm the specificity of the assay. Data shown represent an average of six replicate PCR samples +/− SD. Data are presented as the number of copies of HIV DNA per 100 cells. (C) Three days after spinoculation, cells were either lysed for DNA isolation or stimulated with PMA+ionomycin for 24–72 hours. Peak GFP expression data are shown as the mean of three replicate samples. HIV integration was analyzed by Alu-gag qPCR to specifically detect integrated proviral DNA, and levels were normalized to levels obtained for the single copy RNaseP gene. Data shown are average of six replicate PCR samples +/− SD. Data are presented as copies of integrated HIV DNA/100 cells and the number of GFP+ cells/100 cells.

Unintegrated HIV-1 DNA is unstable and does not represent a major mechanism for long-term persistence of HIV *in vivo* [13], [35] Conversely, stably integrated HIV proviruses can be highly persistent and are responsible for durable forms of latent HIV infection. As such, relevant models of HIV-1 latency must specifically detect reactivation of integrated proviruses and exclude background viral protein production from unintegrated forms. To determine if the detected GFP, luciferase, and mCherry:luciferase signals were derived from integrated proviruses, we examined reactivation levels in the absence or presence of raltegravir, a potent HIV integrase inhibitor [36]. Optimal concentrations of raltegravir were first determined using spinoculation conditions to infect permissive, activated CD4 T cells (Figure S3). Cultures were treated with raltegravir immediately after spinoculation (Figure 1A, pre-treatment). Under these conditions, the luciferase signal after reactivation was almost abolished, validating the potent antiviral activity of raltegravir at the concentration tested. A few GFP^+^ cells were detected after raltegravir pre-treatment, possibly reflecting low-level expression from multiply spliced mRNAs produced by unintegrated viruses [37] (Figure 2A). Next, to determine if the reporter signals emanated from integrated latent proviruses, raltegravir was added immediately before reactivation rather than after spinoculation (Figure 1A, Pre-activation). Under these conditions, levels of reactivation were 20–45% lower than in untreated samples (Figure 2A). These findings suggest that 20–45% of the signal generated during reactivation is from viruses that had not yet completed integration at the time of stimulation. Activation-induced integration and expression of these viruses were inhibited by raltegravir. Conversely, 55–80% of the signal appears to derive from integrated proviruses that are insensitive to raltegravir when added at reactivation. To eliminate the signal contributed by preintegration forms of latent HIV and to inhibit HIV spread in the cultures, raltegravir was routinely added before reactivation in all subsequent experiments.

The insensitivity of the reporter signal to raltegravir at activation strongly argued for the presence of integrated latent proviruses in these cells. To confirm the presence of integrated proviruses, we performed Alu-gag PCR to specifically detect integrated HIV DNA [32] (Figure 2B). An Alu-gag PCR signal was not detected when cells were infected in the presence of raltegravir, AMD3100 or Efavirenz, validating the specificity of the assay for integrated HIV DNA (Figure 2B, left panel). A gag PCR to detect both integrated and unintegrated forms of HIV DNA demonstrated that infection was blocked at the level of integration in the presence of Raltegravir (Figure 2B, right panel). Of note, the amount of integrated HIV DNA varied from donor to donor with a range of 0.65–7.8 copies of integrated HIV DNA per 100 cells (Figure 2C). Levels of GFP expression occurring after reactivation correlated with the frequency of cells harboring integrated provirus (Figure 2C). Interestingly, levels of GFP expression were consistently slightly lower than integration levels, suggesting some cells remain latent after stimulation or cells that respond to stimulation harbor more than one copy of HIV DNA.

### Latently infected cells exhibit robust responses to T-cell activators

**Figure 3:**
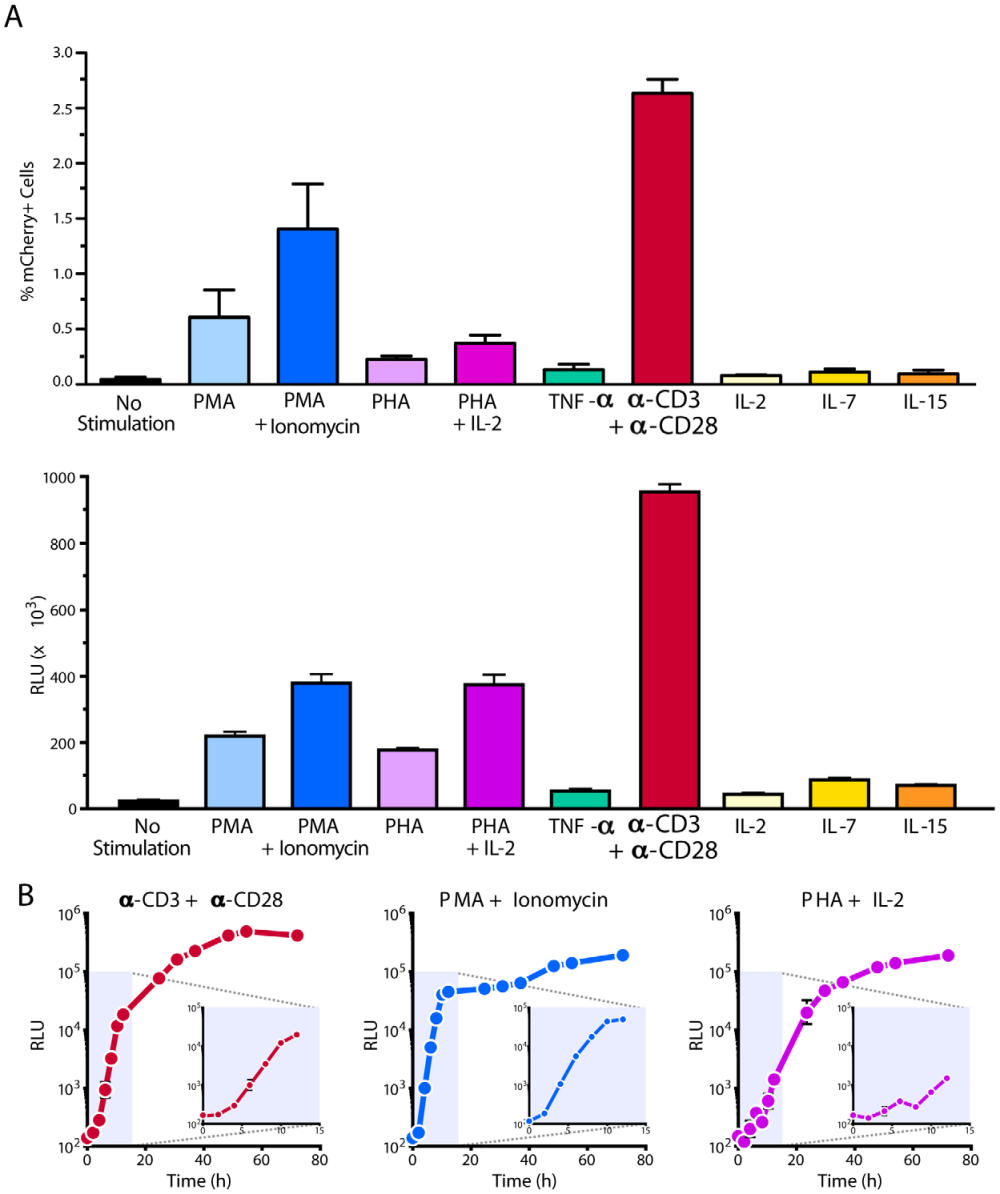
Kinetics of HIV-1 reactivation. (A) Latently infected cells were generated as described in Figure 1 with NL4-3 Luciferase virus or NL4-3 mCherry:Luc virus. Cells were either cultured in the presence of media alone or stimulated with 200 nM PMA, 200 nM PMA and 1.5 µM ionomycin, 10 µg/ml PHA, 10 µg/ml PHA with 100 units/ml IL-2, 10 ng/ml TNF-α, anti-CD3+anti-CD28 beads (ratio 1∶1), 100 units/ml IL-2, 62.5 ng/ml IL-7, or 12.5 ng/ml IL-15. Cells were harvested after 48 h of stimulation. RLU shown were normalized based on total protein present in the various cell lysates. All stimulations were performed in triplicate with error bars representing +/− SD. Results are representative of experiments performed with cells from four independent donors. (B) Latently infected cells from the same individual donor were stimulated with anti-CD3+anti-CD28 beads (ratio 1∶1), 200 nM PMA with 1.5 µM ionomycin, or 10 µg/ml PHA with 100 units/ml IL-2 and harvested at the indicated times post-stimulation. Results are representative of kinetic experiments performed with cells isolated from three independent donors.

Various cellular activating agents stimulate latent HIV proviruses in primary cell models and primary cells from HIV-1-infected individuals [18], [38], [39]. First, uninfected resting CD4 T cells were treated with various concentrations of known cellular activating agents and analyzed for CD25 expression, CD69 expression, and viability to determine optimal activation concentrations (Figure S4 and data not shown). We then screened a panel of activating agents at optimal concentrations for relative efficacy in our model. The most effective agents were, in decreasing order, anti-CD3+anti-CD28 antibodies, PHA+IL-2 and PMA+ionomycin. These findings mirror the most effective agents for reactivating latent virus from patient samples [4], [39], [40] (Figure 3A). The results with replication-competent NL4-3 Luciferase, NL4.3 mCherry:Luc, and GFP-expressing viruses correlated well, although the dynamic range of the response was again greatest with the luciferase reporters (Figure 3 and Figure S5). Of note, TNF-α, a potent activator of HIV-1 latency in many cell line models, was ineffective in this primary cell model. This result mirrors the poor effectiveness of TNF-α when added as a single agent activator in primary patient samples and is consistent with the minimal expression of TNFR1 or TNFR2 on resting CD4 T cells [18], [39], [41], [42]. These findings provide further support for the physiological relevance of this primary CD4 T-cell model of HIV-1 latency (Figure 3A).

### HIV reactivation can be rapidly detected after T-cell stimulation

Kinetic studies of HIV-1 reactivation in primary cells are often hindered by low signals and small sample sizes. To assess the kinetics of proviral reactivation in our model, we selected the three classes of strongest activators: anti-CD3+anti-CD28, PHA+IL-2, and PMA+ionomycin. The stimulators exhibited different kinetics within the first 12 h after reactivation. With the large dynamic range for the NL4-3 luciferase virus, we consistently detected viral reactivation within only 2–6 h (Figure 3B). For PMA+ionomycin with its rapid mechanism of cellular activation, we saw reactivation in cells within 2 h (Figure 3B, see insets). PMA+ionomycin and PHA+IL-2 induced a continuous increase in viral reactivation over 72 h, and anti-CD3+anti-CD28-induced reactivation typically peaked after 54 h. These kinetic results again highlight the robust nature of this T-cell model of HIV-1 latency. The rapid reactivation kinetics with PMA+ionomycin suggests that HIV latency can be rapidly reversed with the appropriate inducing agents and that the process may not require new protein synthesis.

### Strong cellular activators potently reactivate latent proviruses

**Figure 4:**
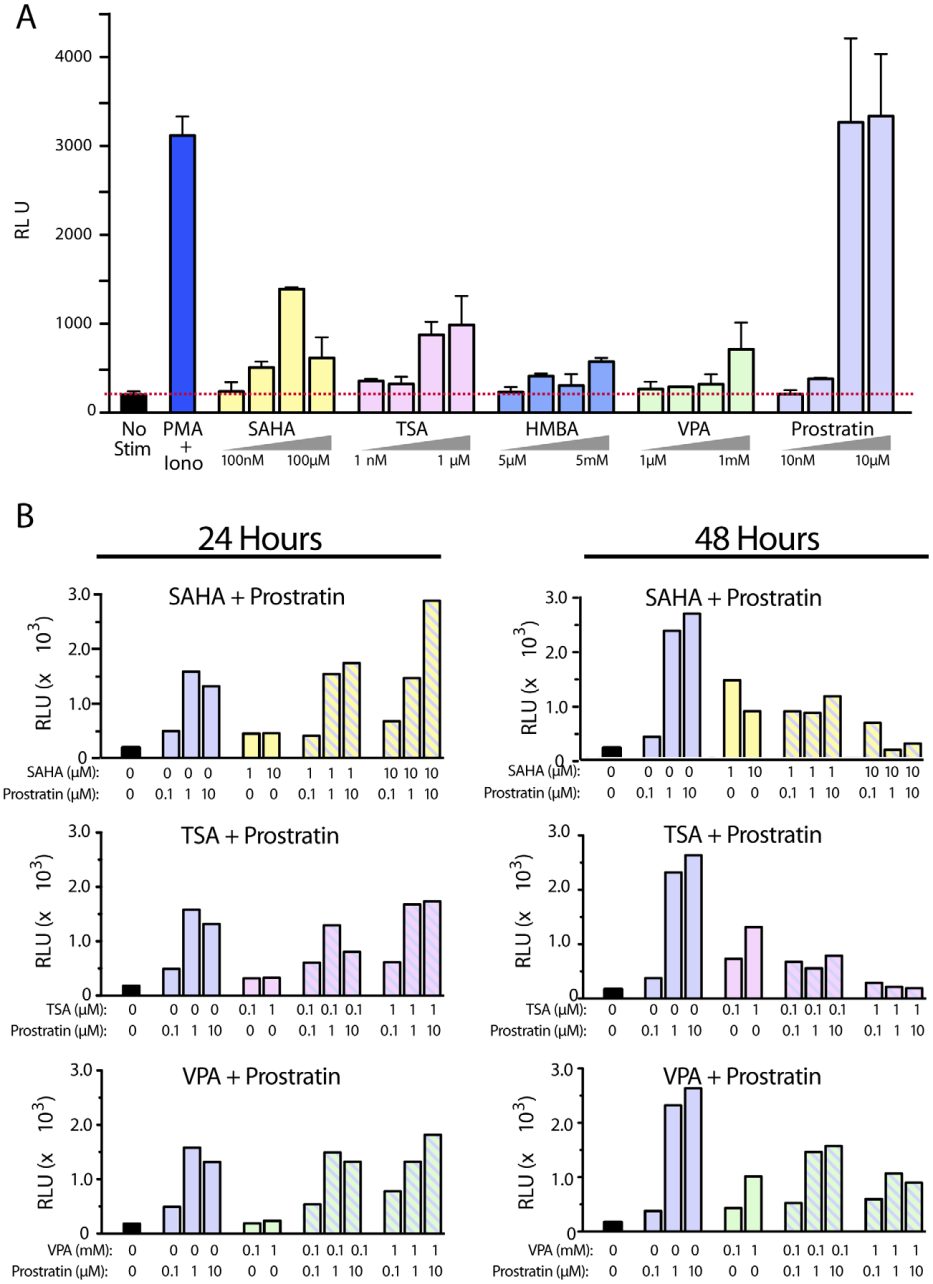
Multiplex screening of inducing compounds on the reactivation of HIV-1 latency. (A) Latently infected cells were stimulated with 10-fold increasing concentrations of SAHA, TSA, HMBA, VPA, or prostratin. Cells were treated with 200 nM PMA+1.5 µM ionomycin as a positive control. The highest and lowest concentrations of the 10-fold dilution series are indicated for each compound tested. Stimulations were performed in triplicate reactions and error bars represent +/− SD. (B) Cells were treated for 24 or 48 h with the indicated concentration of compounds. Results are representative of independent experiments performed with at least three independent donors.

Next, we tested combinations of cellular activating agents for their abilities to activate latent HIV proviruses in the primary CD4 T cells. Specifically, we interrogated agents that induce NF-κB or P-TEFb or that act by promoting changes in chromatin structure surrounding the HIV-1 LTR [11], [18], [43]. Three different histone deacetylase (HDAC) inhibitors (i.e., SAHA, VPA, and TSA) were tested over a 4-log concentration range (Figure 4A). Prostratin was used as a strong activator of NF-κB, and HMBA was tested as a strong activator of P-TEFb. Prostratin added as a single agent matched the potent inducing activity of the combination of PMA+ionomycin. (Figure 4A). One clear drawback with prostratin was its rather narrow dose range (1–10 µM) (Figure 4A,B). SAHA displayed reduced activity at concentrations greater than 10 µM, due mainly to its cellular toxicity (Figure 4A and data not shown). HMBA and VPA exhibited weaker inducing activity in these primary CD4 T cells with effects occurring only in the mM range. These findings further highlight how this primary cell model system can be used as an experimental platform to screen candidate activators and for subsequent dose-ranging studies.

### Transcriptional activators fail to synergize with chromatin-modifying agents

Increasing interest has focused on combinations of inducers to synergistically activate latent proviruses. Multiple mechanisms have been proposed for HIV-1 latency, and simultaneous induction of diverse pathways might be more effective than relying on a single pathway (reviewed in [6]). For example, in cell line models of HIV-1 latency, prostratin and HDAC inhibitors synergistically reactivated latent proviruses [44], [45]. Additionally, reactivation studies in CD8-depleted PBMCs from HAART-treated subjects suggested HDAC inhibitors and transcriptional activators might work together in these cells as well [46].

Using our primary CD4 T-cell model system, we tested combinations of drugs, each at its most effective concentration. Prostratin is a potent stimulator of NF-κB in CD4 T cells. At 1–10 µM, it reactivated HIV-1 latency as effectively as T-cell receptor agonists in every donor evaluated. To determine if additional latent provirus could be reactivated, we evaluated prostratin in combination with HDAC inhibitors. The drug pairings were designed to promote increased latent proviral reactivation by triggering complementary intracellular signaling pathways. Combinations of drugs typically worked somewhat better than single drugs (Figure 4B). Results were consistent when the NL4-3 GFP IRES Nef virus was employed indicating that combinations of drugs also did not achieve a significantly higher proportion of reactivated cells (Figure S6). Modest synergies were seen with some combinations (e.g., 10 µM prostratin+10 µM SAHA), but they were typically transient and disappeared after 48 h (Figure 4B). However, unlike prostratin alone, the effects of the synergies were not consistent among different donors (Figure 4B and Table 1). Prostratin at 1 µM or more was typically as effective as PMA+ionomycin. This finding is consistent with results from other primary T-cell models of latency, suggesting that addition of a strong activator of NF-κB is sufficient for robust HIV-1 reactivation [18], [40], [43] but in contrast to one latency model formed in central memory CD4 T cells where NF-κB inducers were ineffective [15]. Although HDAC inhibitors showed modest viral reactivation in all donors tested, these effects were overshadowed with a strong transcriptional activator.

**Table 1:** Summary of synergistic activity of inducer combinations.

	24 Hours	48 Hours
**Prostratin+TSA**		
Donor 672	**1.2**	<1.0
Donor 216	<1.0	**1.4**
Donor 890	**1.2**	<1.0
**Prostratin+SAHA**		
Donor 672	**1.2**	<1.0
Donor 216	<1.0	**1.7**
Donor 890	**1.9**	<1.0
**Prostratin+VPA**		
Donor 672	**1.2**	<1.0
Donor 216	<1.0	<1.0
Donor 890	**1.4**	<1.0
**HMBA+TSA**		
Donor 672	**1.1**	<1.0
Donor 216	**1.3**	<1.0
Donor 044	<1.0	**1.3**
**HMBA+SAHA**		
Donor 672	<1.0	<1.0
Donor 216	<1.0	**2.1**
Donor 044	**1.3**	**1.2**
**HMBA+VPA**		
Donor 672	<1.0	<1.0
Donor 216	<1.0	<1.0
Donor 044	<1.0	**1.4**

Values are the calculated synergistic index of the inducers when used in combination versus when used as single agents [44]. Each value represents the highest synergistic index value obtained for a given donor and time period of simulation over a range of six dose combinations (prostratin) or four dose combinations (HMBA). Combinations of the following dose concentrations were used: prostratin (0.1, 1, 10 µM); HMBA (0.5, 5 mM); TSA (0.1, 1 µM); SAHA (1, 10 µM); VPA (0.1, 1 mM). The index of synergism was calculated with the following formula: the luciferase value from cells after stimulation with the indicated combination of inducers divided by the sum of the luciferase values from cells after stimulation with each inducer separately. Background luciferase values from unstimulated samples were subtracted prior to synergistic index calculation. Combinations of given inducers that gave a synergistic index >1 are considered synergistic and shown in bolded text.

Next, each HDAC inhibitor was screened in combination with HMBA, which upregulates HIV-1 transcription primarily by activating the P-TEFb complex of cyclin T1 and CDK9 [47]. Again, only modest, transient synergy was observed with any of the combinations (Table 1). Thus, the robust synergistic effects of HDAC inhibitors in combination with prostratin and other agonists in J-Lat cells are not readily translated to these primary CD4 T cells. However, these studies do highlight the utility of this primary cell model of HIV latency for rapid screening of compounds for synergistic activating effects.

### Transitional memory CD4 T cells are preferentially reactivated by NF-κB inducers

Recent studies revealed cellular heterogeneity within the latent reservoir [22], [23]. Specifically, central memory and transitional memory CD4 T cells with latent viruses and likely turnover at different rates [23]. Since our model involves minimal manipulation of the circulating memory CD4 T-cells, we determined if latency was established in both of these cell types and if latent proviruses in each displayed distinguishing patterns of reactivation.

**Figure 5:**
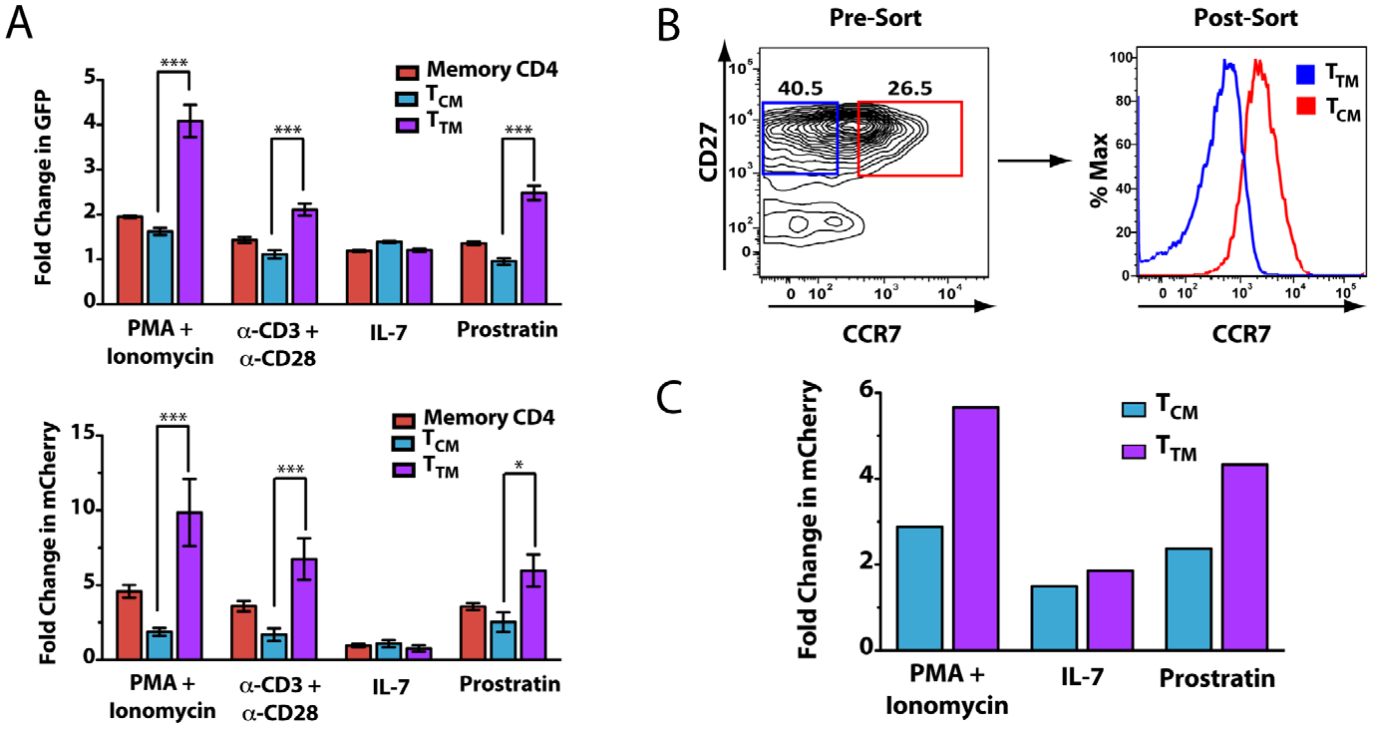
Analyses of reactivation profiles in latently infected transitional memory and central memory CD4 T cells. (A) CD4+CD45RO+ cells were purified by single step negative selection and HIV latency was established in these cells as described above. Cells were plated in 96-well plates and stimulated with 200 nM PMA with 1.5 µM ionomycin, anti-CD3+anti-CD28 beads (ratio 1∶1), 62.5 ng/ml IL-7, 10 µM prostratin, or left unstimulated for 24 h. Cells were stained with CD45RA-APC-H7, CCR7-PE-Cy7, CD27-APC, and CD45RO-FITC (NL4-3 mCherry:Luc) or CD45RO-PE (NL4-3 GFP), and analyzed for receptor expression and viral reporter expression. To obtain fold stimulation ratios, data were normalized as the percentage of cells expressing the viral reporter with the indicated stimulation divided by the % cells expressing the viral reporter in the absence of stimulation. Data shown represent an average of results obtained from four independent donors for each viral construct. Error bars represent +/− SEM. (B) CD4+CD45RO+ cells (upper panel) were sorted for CCR7+CD27+ central memory cells (T_CM_) and CCR7-CD27+ transitional memory cells (T_TM_). Cells were cultured for 2 days and then infected by spinoculation of NL4-3 mCherry:Luc. At the time of infection, cells were analyzed by flow cytometry for receptor expression to determine the relative levels of CCR7 expression in each sorted population (lower panel). (C) Latently infected cells were either left unstimulated or stimulated for 30 h with the indicated inducers. Two independent donors are shown, and fold change was determined as described above for luciferase levels.

Memory CD4 T cells (CD4+CD45RO+) were isolated from PBMC, and post-integration latency was established as described. Cells were activated with a subset of inducers that reproducibly gave the highest signals after reactivation, including PMA+ionomycin, anti-CD3+anti-CD28 antibodies, and prostratin. We also tested IL-7, because of its role in homeostatic proliferation of these cells and its ability to reactivate latent HIV [18], [23], [24]. Total memory cells, central memory (CCR7+CD27+), or transitional memory (CCR7-CD27+) were monitored by flow cytometry, and the percentage of cells within each subset expressing either GFP or mCherry after adding inducers was determined (Figure 5A). The results are presented as fold change in fluorescence between induced and uninduced cells, thereby controlling for differences in background fluorescence in the different cellular subsets. While proviral latency was established in both populations, transitional memory cells (T_TM_) were significantly more susceptible to reactivation with PMA+ ionomycin, anti-CD3+anti-CD28 antibodies, and prostratin as assessed with GFP and mCherry reporter viruses (Figure 5A). Central memory cells (T_CM_) and transitional memory cells (T_TM_) exhibited similar levels of reactivation with IL-7. These findings suggest that these different subsets of latently infected memory CD4 T cells respond differently to strong activators of nuclear NF-κB expression but similarly to IL-7.

Although these results suggest that transitional memory cells may be more susceptible to certain inducers, adding these inducers might have changed the distribution of central and transitional memory cells even in a short experimental time. To address this possibility, we sorted memory cells into transitional and central memory cell subsets (Figure 5). Cells were infected with NL4-3 mCherry:Luc, and HIV latency was established in each. Cells were reactivated (Figure 5A), although the very low numbers of recovered cells did not permit analysis of all inducers with cells from a single donor (Figure 5C). Since these populations are no longer cultured in bulk, we chose to analyze the fold change in luciferase levels after stimulation since this reporter consistently yielded the greatest dynamic range. As in the prior experiments, transitional memory cells were more susceptible to stimulation with PMA+ionomycin, anti-CD3+anti-CD28 antibodies, and prostratin but not IL-7. Importantly, although these cell types exhibited relative differences in the level of reactivation achieved with each inducer, both cell types were reactivated with each inducer.

## Discussion

We report here a novel primary T-cell model of HIV-1 latency. Our model has several advantages over existing models [7], [20], [21]. Its speed and reproducibility facilitate the screening of unknown compounds and unique combinations and concentrations of known activators. The novel mCherry-luciferase dual reporter virus allow us to assess the number of cells responding to a specific inducer (mCherry) and the magnitude of the response within the entire population (luciferase). The single-step negative purification step for CD4 T cells from peripheral blood minimizes the manipulations of cells. We estimate that 200–1000 reactivation conditions can be screened with cells from a single unit of blood. The high signal-to-noise ratio with the luciferase reporter viruses suggests that non-optimized inducers with low reactivation activity can be readily detected. Thus, this assay could be valuable in the search for novel inducers or combinations of inducers.

Cells respond in many ways when HIV-1 proteins are expressed at high levels, causing differences in internal and external signaling properties. Our kinetic studies revealed viral proteins can be detected in latently infected cells within 2 h after induction. The full-length, replication-competent virus in this system also closely mimics latent HIV-1 infection *in vivo* and could be useful for monitoring potential changes in cellular responses associated with reactivating latent proviruses and expressing viral proteins. Of note, Nef+ and Nef− viruses responded to activators with similar kinetics, suggesting that Nef may not be important after viral reactivation. Effectiveness of “purging” of the latent reservoir can also be monitored in this system since cells are infected with a cytopathic and replication-competent virus. Finally, the release and accumulation of viral particles after reactivation can be quantified, thus providing useful information on the efficacy of various stimuli.

Using this model, post-integration HIV latency can be rapidly and reproducibly established. Our findings indicate that HIV integration levels correlate well with the levels of HIV expression observed after cellular stimulation [24]. However, even with the most potent inducers, HIV reactivation levels reflect only a fraction of the total integrated HIV DNA detected. This finding suggests a variegated response within the entire population of latently infected cells with each cell likely containing a single integrated provirus. However, we cannot completely exclude the possibility that some cells contain more than one provirus, although the frequency of such an event is likely to be very low. Overall, we believe these results are comparable to results in patient samples where approximately 99% of the proviral DNA cannot be detected by limiting dilution co-culture growth assays [1] or where only a fraction of J-Lat CD4 T cells each containing a single provirus respond to inducers [12]. This model may also be useful for further characterizing the subset of latently infected cells that fail to respond to classic reactivation signals to discern the underlying mechanism(s).

Numerous model systems and data generated with patient-derived cells suggest that NF-κB is important in reactivating HIV-1 from latency [18], [38], [39], [40], [43], [48], [49]. However, another highly robust primary CD4 T-cell model of HIV-1 latency did not agree [15]. Although our findings suggest that CD4 memory T-cell subsets achieve different levels of activation with various inducers, NF-κB appears to be involved. Interestingly, latently infected transitional memory CD4 T cells preferentially responded to prostratin, a strong inducer of NF-κB but not NFAT [48]. Model systems that more closely resemble a central memory CD4 T-cell phenotype might be less dependent on NF-κB for viral reactivation [15]. Nevertheless, our findings indicate that all inducers reactivate HIV in each of these memory subsets, although the magnitude of reactivation appears greater in transitional memory CD4 T cells. We believe this primary model system will prove useful for continuing to dissect the curious differences between the two memory cell populations.

The precise mechanism by which the latent reservoir is established and maintained *in vivo* is an area of ongoing debate. More studies are needed to determine the relative contributions of different cellular latent reservoirs to ongoing viremia during therapy and viral rebound after cessation of therapy [50], [51], [52]. A heterogeneous latent reservoir could complicate development of effective eradication strategies aimed at purging the latent reservoir. One of the latently infected cell types identified, transitional memory CD4 T-cells, are latently infected *in vivo* and may be maintained by homeostatic proliferation despite prolonged antitretroviral therapy [23]. If these latently infected cells could be specifically targeted *in vivo* it is possible that other latent reservoirs might naturally decay over time [22]. Our results demonstrate that these transitional memory CD4 T cells may be easily targeted by T-cell activators, including prostratin. Although additional studies focusing on dissecting the different reactivation properties of these discrete latently infected cell populations are urgently needed, the model system presented here provides the flexibility to begin identifying optimal reactivation strategies.

One strategy to purge the latent reservoir involves cytokines or small molecules to attack different molecular pathways that maintain latency. Several studies suggested that combinations of NF-κB inducers (e.g., prostratin or PMA) and HDAC inhibitors (valproic acid or trichostatin A) might act synergistically [44], [45], [46]. We observed modest synergy with some combinations of activators. However, in agreement with previous reports, this synergy was often transient and lacked consistency between different donors [45], [46]. Our results in this primary CD4 T-cell model suggest that prostratin alone may be nearly as potent as this agent in combination with HDAC inhibitors. Donor-dependent differences in the synergistic activation observed with combinations of prostratin and SAHA, if confirmed in patients, would dampen enthusiasm for this approach. The chromatin environment might have a more significant role in establishing latency in proliferating cell lines than in quiescent primary CD4 T cells. Additionally, the activation and binding of strong transcription factors to the HIV LTR could interrupt RNA Pol II transcriptional interference from upstream promoters, a process that is known to help maintain HIV latency [53], [54], [55]. Our findings certainly raise the possibility that non-toxic single agents might prove capable of mounting a strong attack on the latent reservoir.

One very important unanswered question in the field is which of the primary CD4 T-cell models most closely recapitulates the biology of HIV latency occurring *in vivo*. While our model has several attractive features including the ability to rapidly establish latency in specific memory CD4 T-cell subsets and to test the effects of inducers on these cellular reservoirs, it will be important to test this model side-by-side with others. Only by carefully comparing results from the different models to results obtained with cells isolated from HIV-infected patients on HAART will it be possible to identify the best *in vitro* models for *in vivo* HIV latency.

As new translational approaches for eliminating the latent reservoir emerge, a flexible, high-throughput, and highly reproducible model of latent HIV-1 infection becomes increasingly important. The versatility of this primary cell model could make it useful for studies ranging from high-throughput compound screening to molecular characterization of the mechanisms of HIV-1 latency to studies of reservoirs within different memory CD4 T-cell subsets. We hope that this model will help overcome a major barrier in the HIV latency field allowing the rapid acquisition of data previously considered unobtainable.

## Supporting Information

Figure S1 **(a) Uninfected peripheral blood cells were purified by one-step negative selection for either total CD4 T cells or CD45RO+ CD4 memory T cells.** 24 hours after isolation uninfected cells were either stained with CD4-FITC and CD3-APC or CD45RO-FITC and CD45RA-APC and analyzed by flow cytometry. **(b) Reactivation profiles of cells latently infected with NL4-3 luciferase.** Latently infected cells were cultured with media alone or media containing 200 nM PMA and 1.5 µM ionomycin and harvested after 48 hours of culture. Luciferase levels are reported as relative light units (RLU) and have been normalized to total protein content in cell lysates to control for different cellular proliferation rates. **(c) Uninfected peripheral blood cells were purified by one-step negative selection for either total CD4 T cells or resting (CD25-/CD69-/HLA-DR-) CD4 T cells.** Cells were infected and cultured as described in (b).
(TIF)

Figure S2 **Reactivation profiles of cells latently infected with NL4-3 luciferase.** Latently infected cells generated from 10 representative uninfected donors were cultured with media alone or media containing 200 nM PMA and 1.5 µM ionomycin and harvested after 48 hours of culture. Luciferase levels are reported as relative light units (RLU) and have been normalized to total protein content in cell lysates to control for different cellular proliferation rates.
(TIF)

Figure S3 **CD4 T cells were activated for 3 days prior to infection.** Activated cells were infected by spinoculation with NL4-3 GFP virus as described above. Immediately after spinoculation, cells were washed three times and cultured for 48 hours in the absence of drug or in the presence of the indicated concentration of raltegravir or 118-D-24. Cells were evaluated for GFP expression 48 post-infection. 100% infection was scored as the percentage of GFP+ cells obtained in the absence of drug.
(TIF)

Figure S4 **Uninfected CD4 T cells were treated for 48 hours with PHA alone (10 and 5 µg/ml), IL-2 alone (500, 100 and 10 U/ml) or PHA and IL-2 in combination at indicated concentrations.** Cells were analyzed by flow cytometry to determine the percentage of cells expressing CD25 or CD69 (a) and cell viability (b). Based on activation marker expression and viability, optimal concentrations were determined to be 10 µg/ml for PHA alone, 100 U/ml for IL-2 alone, 10 µg/ml/100 U/ml for PHA+IL-2.
(TIF)

Figure S5 **Latently infected cells were generated as described in** **Figure 1** **with NL4-3 GFP virus.** Cells were either cultured in the presence of media alone or stimulated with 200 nM PMA, 200 nM PMA with 1.5 µM ionomycin, 10 µg/ml PHA, 10 µg/ml PHA with 100 units/ml IL-2, 10 ng/ml TNF-α, anti-CD3+anti-CD28 beads (ratio 1∶1), 100 units/ml IL-2, 62.5 ng/ml IL-7, or 12.5 ng/ml IL-15. Cells were harvested after 48 hours of stimulation and GFP was analyzed by flow cytometry. All stimulations were performed in triplicate with error bars representing +/− SD. Results are representative of experiments performed in 3 different donors.
(TIF)

Figure S6 **Cells infected with NL4-3 GFP were treated for 24 hours with the indicated concentration of compounds.** Viability (right panels) and reactivation profiles (left panels) are representative of independent experiments performed with at least 3 independent donors.
(TIF)
