# Supplementary figures and images for: A Flexible Model of HIV-1 Latency Permitting Evaluation of Many Primary CD4 T-Cell Reservoirs
Source: PLoS One. 2012 Jan 24;7(1):e30176. doi: 10.1371/journal.pone.0030176 (PMC3265466; doi:10.1371/journal.pone.0030176)

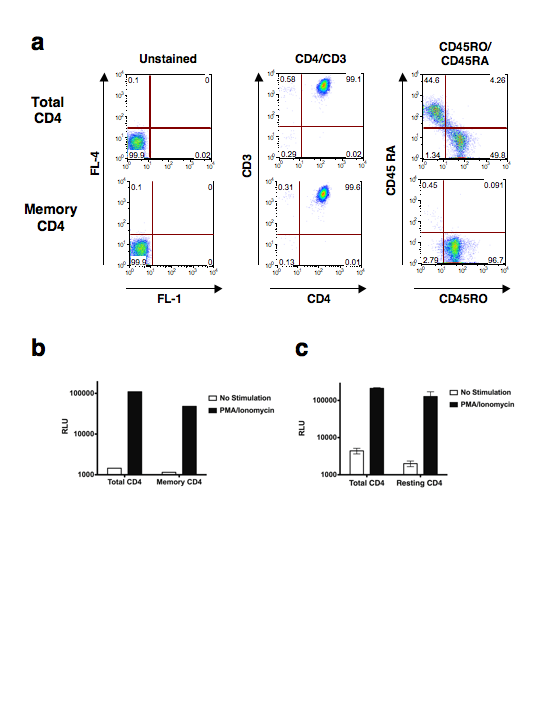

Supplement: Figure S1 — (a) Uninfected peripheral blood cells were purified by one-step negative selection for either total CD4 T cells or CD45RO+ CD4 memory T cells. 24 hours after isolation uninfected cells were either stained with CD4-FITC and CD3-APC or CD45RO-FITC and CD45RA-APC and analyzed by flow cytometry. (b) Reactivation profiles of cells latently infected with NL4-3 luciferase. Latently infected cells were cultured with media alone or media containing 200 nM PMA and 1.5 µM ionomycin and harvested after 48 hours of culture. Luciferase levels are reported as relative light units (RLU) and have been normalized to total protein content in cell lysates to control for different cellular proliferation rates. (c) Uninfected peripheral blood cells were purified by one-step negative selection for either total CD4 T cells or resting (CD25-/CD69-/HLA-DR-) CD4 T cells. Cells were infected and cultured as described in (b). (TIF) [file pone.0030176.s001.tif]

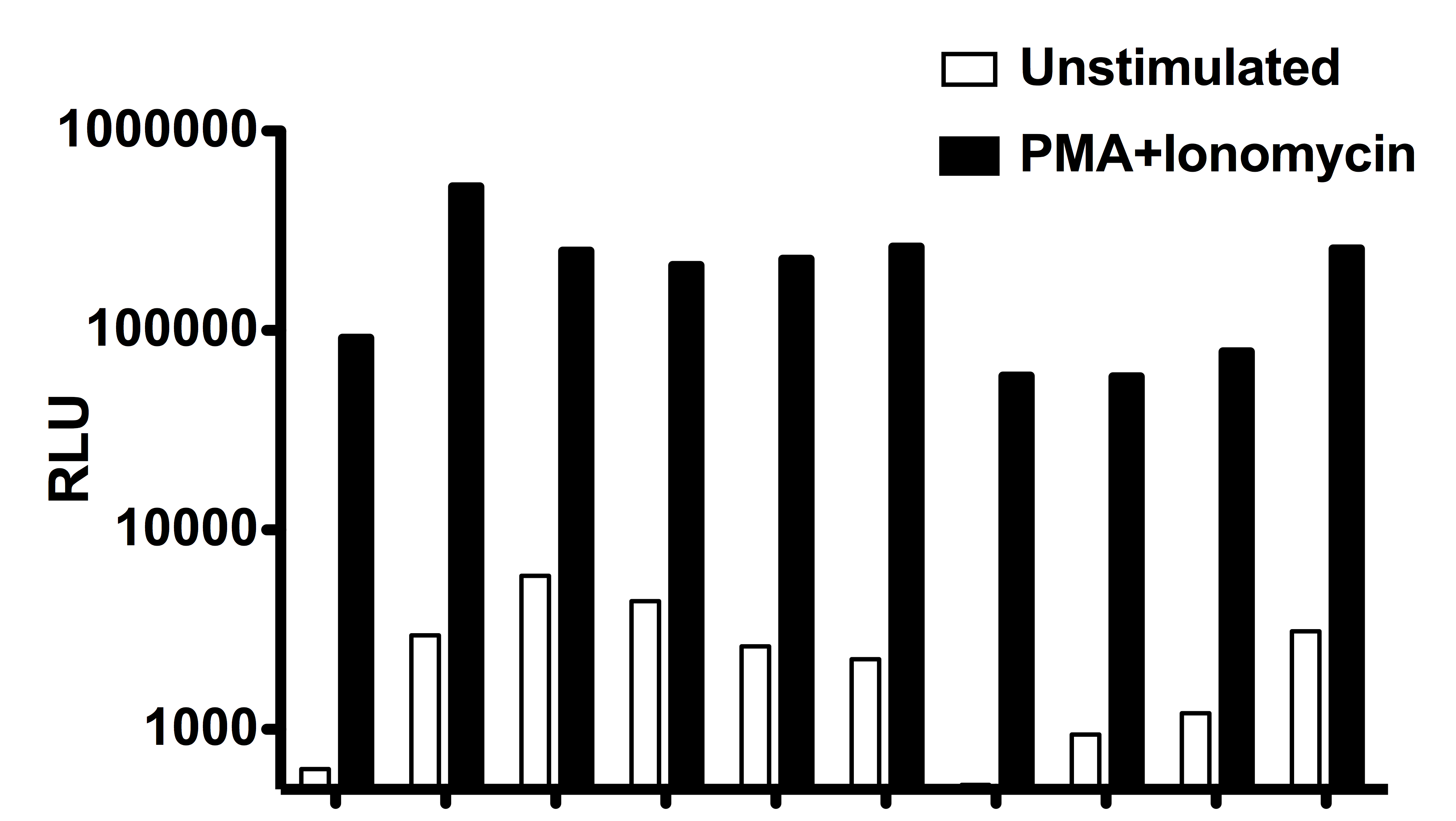

Supplement: Figure S2 — Reactivation profiles of cells latently infected with NL4-3 luciferase. Latently infected cells generated from 10 representative uninfected donors were cultured with media alone or media containing 200 nM PMA and 1.5 µM ionomycin and harvested after 48 hours of culture. Luciferase levels are reported as relative light units (RLU) and have been normalized to total protein content in cell lysates to control for different cellular proliferation rates. (TIF) [file pone.0030176.s002.tif]

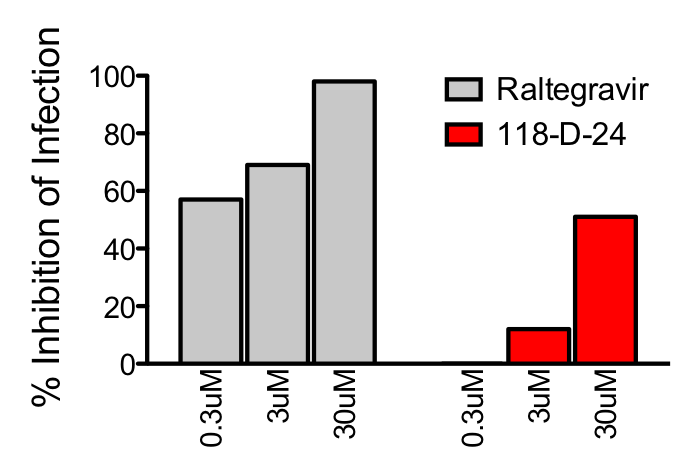

Supplement: Figure S3 — CD4 T cells were activated for 3 days prior to infection. Activated cells were infected by spinoculation with NL4-3 GFP virus as described above. Immediately after spinoculation, cells were washed three times and cultured for 48 hours in the absence of drug or in the presence of the indicated concentration of raltegravir or 118-D-24. Cells were evaluated for GFP expression 48 post-infection. 100% infection was scored as the percentage of GFP+ cells obtained in the absence of drug. (TIF) [file pone.0030176.s003.tif]

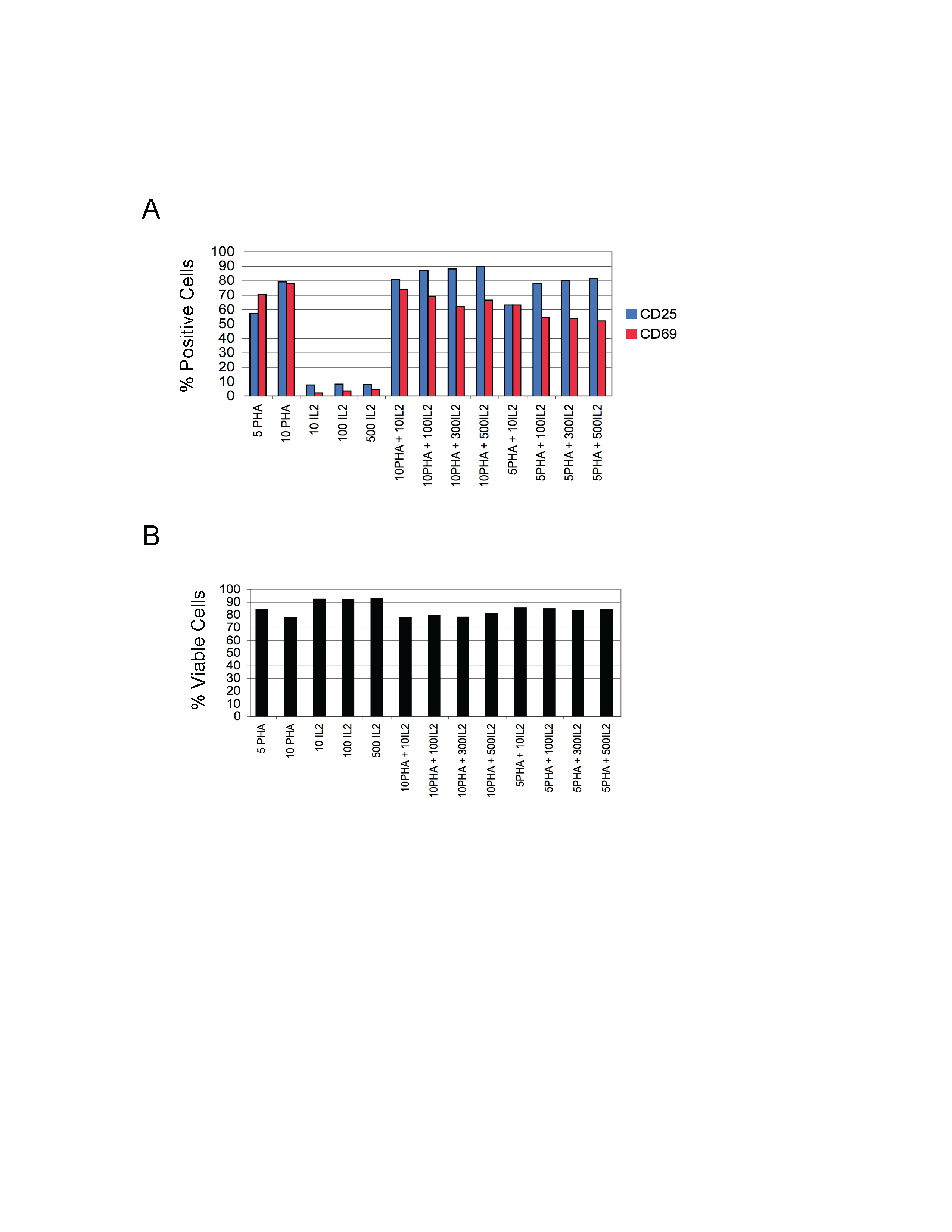

Supplement: Figure S4 — Uninfected CD4 T cells were treated for 48 hours with PHA alone (10 and 5 µg/ml), IL-2 alone (500, 100 and 10 U/ml) or PHA and IL-2 in combination at indicated concentrations. Cells were analyzed by flow cytometry to determine the percentage of cells expressing CD25 or CD69 (a) and cell viability (b). Based on activation marker expression and viability, optimal concentrations were determined to be 10 µg/ml for PHA alone, 100 U/ml for IL-2 alone, 10 µg/ml/100 U/ml for PHA+IL-2. (TIF) [file pone.0030176.s004.tif]

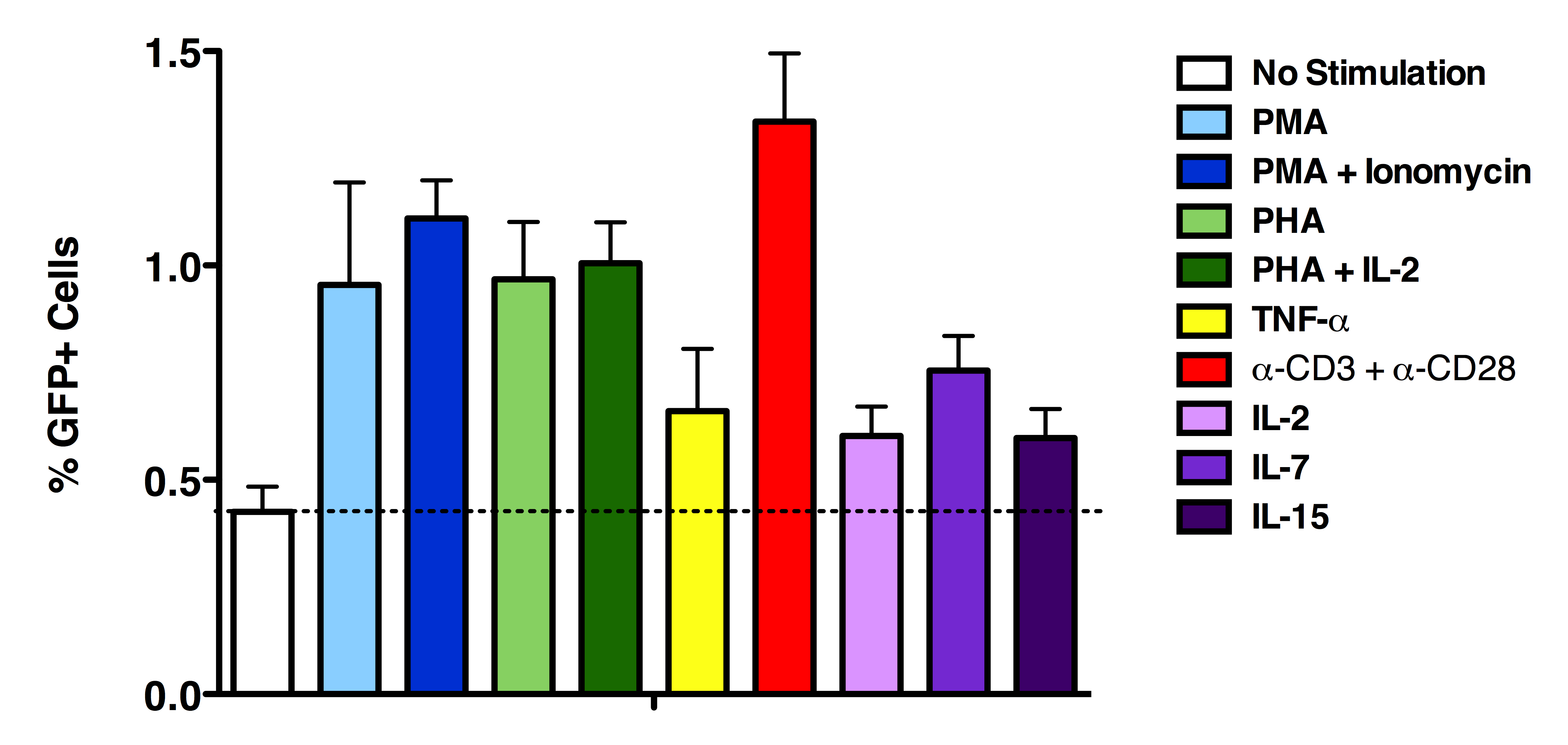

Supplement: Figure S5 — Latently infected cells were generated as described in Figure 1 with NL4-3 GFP virus. Cells were either cultured in the presence of media alone or stimulated with 200 nM PMA, 200 nM PMA with 1.5 µM ionomycin, 10 µg/ml PHA, 10 µg/ml PHA with 100 units/ml IL-2, 10 ng/ml TNF-α, anti-CD3+anti-CD28 beads (ratio 1∶1), 100 units/ml IL-2, 62.5 ng/ml IL-7, or 12.5 ng/ml IL-15. Cells were harvested after 48 hours of stimulation and GFP was analyzed by flow cytometry. All stimulations were performed in triplicate with error bars representing +/− SD. Results are representative of experiments performed in 3 different donors. (TIF) [file pone.0030176.s005.tif]

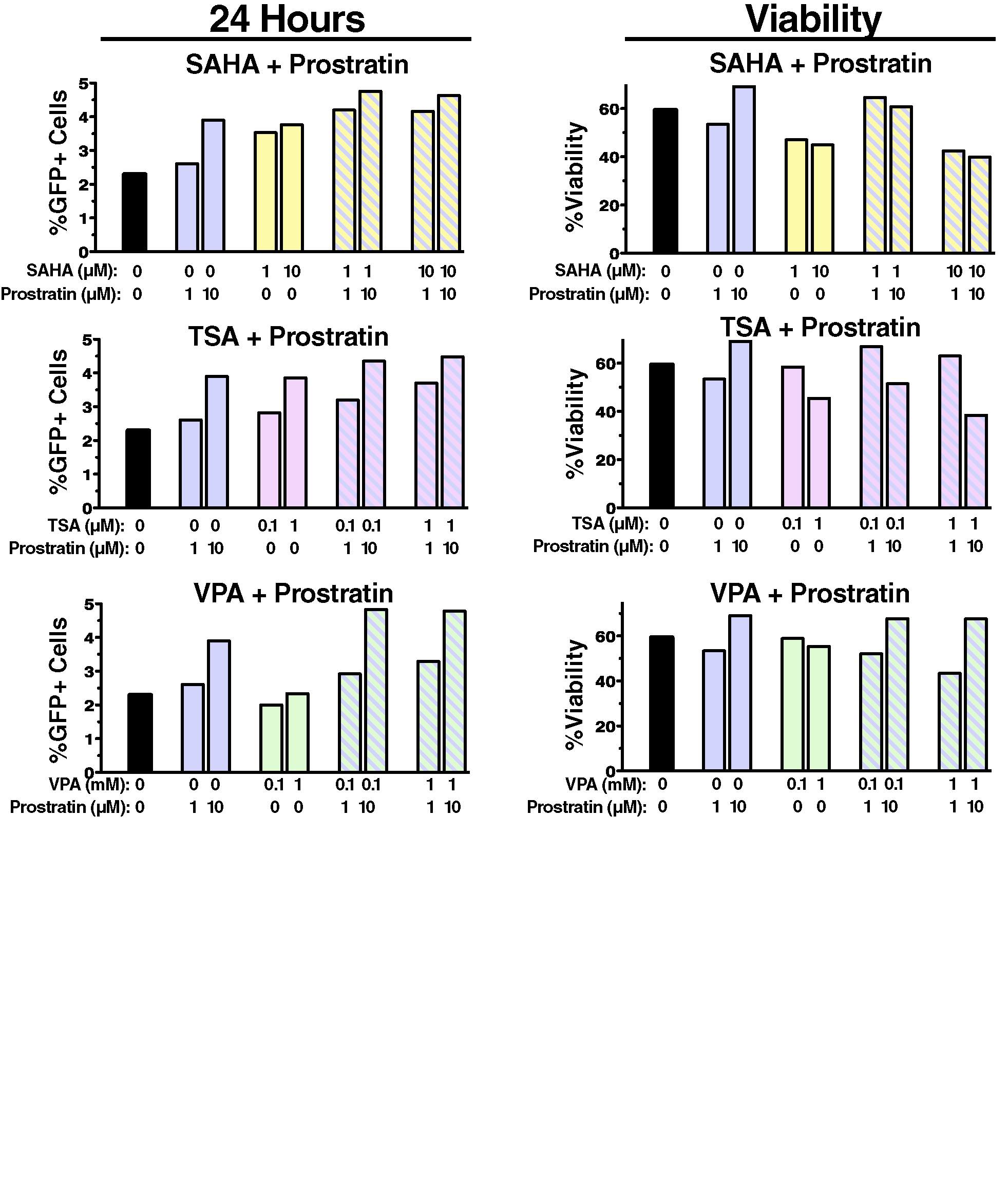

Supplement: Figure S6 — Cells infected with NL4-3 GFP were treated for 24 hours with the indicated concentration of compounds. Viability (right panels) and reactivation profiles (left panels) are representative of independent experiments performed with at least 3 independent donors. (TIF) [file pone.0030176.s006.tif]
